# Integration of chronic disease prevention and management services into primary care (PR1MaC): findings from an embedded qualitative study

**DOI:** 10.1186/s12875-018-0898-z

**Published:** 2019-01-09

**Authors:** Martin Fortin, Maud-Christine Chouinard, Bayero Boubacar Diallo, Tarek Bouhali

**Affiliations:** 1 Département de médecine de famille et de médecine d’urgence, Université de Sherbrooke, Sherbrooke 3001, 12e avenue Nord, Sherbrooke, Québec J1H 5N4 Canada; 2 Centre intégré universitaire de santé et de services sociaux du Saguenay–Lac-Saint-Jean, Hôpital de Chicoutimi, 305, Saint-Vallier, Chicoutimi, Quebec G7H 5H6 Canada; 3 Département des sciences de la santé, Université du Québec à Chicoutimi, 555, boulevard de l’Université, Chicoutimi, Québec G7H 2B1 Canada

**Keywords:** Chronic diseases, Primary health care, Intervention, Disease management, Qualitative research, Program evaluation

## Abstract

**Background:**

The PR1MaC study was conducted to evaluate the integration of Chronic Disease Prevention and Management services into primary care practices and was reported effective. The aim of this study was to further explore the effects of the PR1MaC intervention on patients and their family.

**Methods:**

We conducted a qualitative study embedded in a randomized controlled trial. The trial was implemented in eight primary health care practices in the Saguenay region, Quebec, Canada. The interdisciplinary patient-centred team-based intervention included self-management support and a motivational approach. We conducted focus groups and semi-directed individual interviews with patients, family members and healthcare professionals.

**Results:**

Perceived positive effects can be grouped into six major themes: awareness, improved knowledge, improved motivation and empowerment, adoption of healthy behaviours, improvement of health status and improvement of quality of life*.* On the negative side, some participants reported lack of sustainability of newly acquired benefits in the months following the intervention.

**Conclusions:**

Integrating chronic disease prevention and management services into primary care settings had impacts on patients and their family members. These findings are consistent with findings that were reported in the quantitative study. Further studies should address longterm sustainabilility in terms of benefits for the patients.

**Trial registration:**

ClinicalTrials.gov, no.: NCT01319656.

**Electronic supplementary material:**

The online version of this article (10.1186/s12875-018-0898-z) contains supplementary material, which is available to authorized users.

## Background

Primary care is where the majority of patients with chronic diseases and their family members seek care for their multiple chronic conditions [1]. Primary care practices face the challenge of providing high quality care for all patients and chronic conditions on a long-term basis. Integrating chronic disease prevention and management (CDPM) programs into primary care provides an opportunity to enhance the care of patients with chronic diseases directly in the setting where they receive their comprehensive care, while ensuring continuity [2]. CDPM programs by interdisciplinary teams are usually designed to improve outcomes for patients with chronic diseases (CD) such as self-management, adherence to medications, disease specific outcomes, quality of life or use of health care services. The PR1MaC demonstration project consisted of the integration of CDPM services into eight primary care practices in the Saguenay region, Province of Quebec, Canada [3]. The clinical intervention was developed according to the needs expressed by primary care providers, managers and decision-makers consulted prior to its deployment. CDPM services by an interdisciplinary team were added to resources already in place in the practices. Adult patients were referred by their primary care provider to the team to receive an individualized intervention. To participate to the trial, patients had to be between 18 and 75 years of age and present with at least 1 of the following chronic conditions or risk factors: diabetes, cardiovascular disease, chronic obstructive pulmonary disease, asthma, tobacco smoking, obesity, hyperlipidemia, prediabetes, sedentary lifestyle or any combination of these. Patients were required to be fluent in written and spoken French. The key elements of the intervention were: self-management support, patient-centered care, motivational approach, interprofessional collaboration and an individualized care plan. The intervention mean time was 220 min (Standard deviation (SD): 77) with a mean of 2.4 visits (SD: 1.2) over a 3 month period with trained professionals (nurse, nutritionist, kinesiologist, respiratory therapists, smoking cessation therapists) to respond to the objectives identified with the patient at the first encounter with Pr1MaC nurse. All patient encounters were on a one-to-one and face-to-face basis. Details on the intervention are provided elsewhere and summarized in the Additional file 1 [3, 4].

PR1MaC encompassed a pragmatic randomized trial with a delayed intervention group to evaluate the quantitative effects of CDPM services on patients, and a qualitative evaluation of the intervention. In the pragmatic randomized trial [4], the intervention was associated with several improvements for patients: adoption of healthy lifestyle (increased fruit and vegetable consumption, increase in physical exercise), improved emotional well-being, improved self-management and improved knowledge about the management of chronic conditions. A total of 326 patients participated to the trial. The aim of the present study was to further explore the effects of the PR1MaC intervention on patients and their family from their perspectives and those of the healthcare professionals involved in the PR1MaC intervention.

## Methods

### Design

This descriptive qualitative study [5], allowed to comprehensively describe participants’ experience while minimizing researcher bias during analysis. Qualitative approaches embedded in randomized trials can be useful to identify unexpected causal mechanisms or effects [6, 7] in order to deepen our understanding, to gain the perspective of the patients receiving the intervention [8] and to corroborate the quantitative evaluation results.

### Participants and recruitment

This study recruited three types of participants: patients, patients’ family members, and Pr1MaC professionals. From November 2012 to March 2013, patients having completed the intervention were recuited from the PR1MAC trial using a maximum variation sampling [9] regarding age, gender, socio-economic status and participating practices. They were contacted by phone and offered to extend their participation with this optional qualitative inquiry. Family members were identified using indirect solicitation involving the patients. Pr1MaC professionals were contacted by phone at their working place.

### Data collection

Focus groups were conducted with patients 12 months after the intervention. Semi-directed face-to-face individual interviews were conducted with PR1MaC professionals in the same period. In addition family members of the patients where encountered in dyads or single interviews. The interview guides included open questions relating the perceived outcomes of the intervention developed by the research team based on the logic model accessible in the published protocol [3]. The questions were pilot tested with representatives of the different participant types. Interview guides also included questions related to the program implementation, and satisfaction regarding the program. Interviews and focus groups where conducted by two experienced research professionals. The duration of the interviews and focus group was between 30 min to 1 h. Interviews, focus groups and observer notes were audio-recorded and transcribed verbatim. Data were collected as planned even when saturation was achieved in the focus groups with patients and individual interviews with professionals.

### Data analysis

The data collected from all participants (patients, family members and professionals) were analyzed using content analysis. Following an inductive approach combined with thematic analysis, these analyses were done in two steps to identify emerging themes and trends. The first step consisted of reading and analyzing the corpus. The NVivo qualitative data management software (NVivo 9.0, QSR Int. USA) was used to identify units of meaning that were subsequently grouped into nodes of information related to the same topic. The second step consisted of sorting and reviewing the coded information into categories and themes according to different contexts [9]. Two research professionals (one is co-author / BBD) performed and validated the coding under close supervision of a senior author (MCC). Credibility and trustworthiness of the analysis was ensured by using audio-recording and verbatim transcripts, by combining independent and team analysis and by triangulation with the quantitative results. This study is presented following the COREQ reporting standards (Consolidated Criteria for Reporting Qualitative Research) [10].

## Results

### Characteristics of patients

Thirty-six patients accepted to participate to the qualitative study. In order to recruit this number, we had to contact 149 patients from the trial. The main reasons for refusal were: lack of time, lack of interest, health issues and personal reasons. Five family members invited by the partipating patients accepted to participate: one was interviewed and the others were seen in dyads. All 16 invited healthcare professionals accepted to participate to the interviews.

The details of the participants are described in the Table 1. Patients with multimorbidity defined as 3 chronic conditions or more, were predominantly represented in this study which is consistent with the participants of the trial [4]. Mean age of the patients was 58 in they had a mean of 5.9 chonic conditions.

**Table 1:** Participants of the evaluation, by interview type (*n* = 57)

Participants	Type of interview		
	Number of individual interviews	Number of focus groups	Number of participants
Patients		7	36
Family members	1	2	5
PR1MaC professionals: nurses, nutritionists, smoking cessation therapists respiratory therapists	16	0	16

## Findings

Participants reported that the intervention had many positive effects on patients and their family members. Some negative effects were also identified. The following section presented the findings in more details. To facilitate the reading, all the quotes have been regrouped in one single table (Table 2).

**Table 2:** Themes and illustrative quotes

Theme	Illustrative quotes numbered	
Positive effects of the intervention		
*Awareness*	1	It got to me. (Patient #1, transcript #8, focus group)
	2	It made me realize that eating well is important. (Patient #1, transcript #7, focus group)
	3	Before, I would grab fries automatically. (Patient #1, transcript #5, focus group)
	4	The program has turned yellow traffic lights on in the heads of people and prompted them to react. (PR1MaC Nutritionist, transcript #5, individual interview)
	5	There was a patient, his children were overweight. We could see him becoming aware. He said: The kitchen pantry, we’re gonna change it. He was really changing their diet, decreased cookie consumption, juice… we could really see the differences. (PR1MaC Nutritionist, transcript #4, individual interview)
*Improved motivation and empowerment*	6	They gave me back control over my life, it’s not complicated, I’m the boss so I decide. So what comes in [food], I decide. (Patient 3, transcript #9, focus group)
	7	We were aware, we were already careful about food. We worked out a lot. Yes, it didn’t change our health behaviour; our health behaviours were already good. It just reinforced them a bit. (Family member 1, focus group #2)
*Improved knowledge*	8	For me, it helped. Portions. They also taught me what went with what, I really liked that. Then a kinesiologist, it was the first time I met one. She told me how to do my exercises. (Patient 2, focus group #1)
	9	The patient came in with her suitcase full of recipe books. Actually, she needed support. So I took the books one by one. No, that one you can put aside. This one is good. She completely changed her dietary habits after that. (PR1MaC nutritionist #2)
	10	There were things that I knew, but when my doctor told me I had asthma: take this inhaler, this way, but that was it. While with the respiratory therapist, I received plenty of tips, good support, answers to my questions. (Patient 3, focus group #1)
*Adoption of healthy lifestyle habits*	11	It’s not obvious to stop eating salt, cheese, butter. I didn’t know about nutritionists. My encounters with her were worth it. She really helped me make a plan and a menu that wasn’t complicated. I had to put something into it too. At the end of three months, I could see lots of improvement. (Patient 2, focus group #5)
	12	It wasn’t the exercise side for him. We already have a regular exercises routine. It’s really on the nutrition side to try and control... stabilise his cholesterol. So it helped. We learned a lot. How much salt, maximum, you need in a day. We write down our menus and we look at them closely. Oh no! Too much fat, let’s try something else. We added fruit instead of having a second serving or a bigger plate. (Family member 1, focus group #6)
	13	Yes, change is huge, instead of staying put and watching TV, we do activities. That is the big difference, that’s life. (Family member, interview #7)
	14	I told the nurse that I had never made any effort for that. I don’t want a diet or to meet a nutritionist. So she told me. Let’s make a deal. I said yes. Then, I started walking stairs. But I was gaining weight. Yet, often, I would just have supper. Then she said, yes, but you eat poorly. If you could eat breakfast, lunch and dinner, you’ll lose all the weight. I didn’t believe her. Then I lost 30 pounds in three months. (Patient 1, focus group #1)
*Improvement in health status*	15	In general it was successful; either an improvement in their cholesterol levels or an improvement in their diabetes or weight loss, depending on what we were working on. (PR1MaC nutritionist, interview #6)
	16	I lost 40 pounds since September. It was really beneficial for me. The operation, forget that. They can call, but I won’t go. (Patient 1, focus group #4).
*Improvement in quality of life*	17	I continue to exercise. I don’t even run my errands by car, to go pay for my phone, I walk. For me this year, it passed free. It’s funny, no cold to this day, I am happy with that. (Patient 3, focus group #5)
	18	I saw it, maybe at the second follow-up, the patient had changed. And I’m sure that even physically he had improved self-esteem. And I could feel it… the man would come to his encounters happy and proud of himself. (PR1MaC Nutritionist, interview #3)
*Effects on family members*	19	We saw young patients that lived with their parents, who brought documents back home. They showed them. It was the mother who cooked; she took that in hand so her child could eat better, but indirectly she makes everyone eat well. There were spouses too who engaged in that, so good support with their husband or wife, whatever. So it was a family investment. (PR1MaC Nurse, interview #4)
	20	We thought we were good because he had lost 40 pounds, I lost 13. And I didn’t exercise like he did. (Family member 2, focus group #2)
Negative effects of the intervention		
	21	I’m quite happy; I was successful in changing their dietary habits. For sure I would have liked to do a longer follow-up with them. Yes, it’s good, at the beginning we are motivated but after, what happens after three, 4 months. I think that when we are let loose all alone in nature, it’s not as easy, especially when vacations come up. So I think a longer follow-up is necessary. (PR1MaC Nutritionist, interview #1)
	22	I thought it was a nice approach. But at some point, it’s over. But then, you know, I need a bit more, you know, motivation. (Patient 3, focus group #4)
	23	Yes, yes, that’s what’s missing, follow-up [long term]. (Patient 3, focus group #4)
	24	I started at 185, went up to 195 pounds. Oh, is there a problem? I’m on the PR1MaC diet and I just gained 10–15 pounds. Then I gained even more, I reached 22 pounds. There’s a problem, I am going to stop that diet, it’s urgent now. (Patient 3, focus group #3)
	25	Well, he gave up. He didn’t come [to the encounter] because he gained weight. He wasn’t OK with that. I’m sure. I said, you should go; he said no. But I’m sure that he didn’t come because he had gained weight. (Family member 2, focus group #2)
	26	It’s about motivation, well there are things that we don’t know, and there are things I would like to learn with the nutritionist. I find that I’m not losing weight anymore and that irritates me. (Patient 3, focus group #4)
	27	We have lots of good results. But certain people we did not engage, we would see them in follow-ups and nothing had changed. It didn’t have any impact on their life. (PR1MaC nurse, interview #4)
	28	We don’t pay enough attention to food. The mother in law cooks traditional foods, she makes dinner. She is used to using a lot of salt, pepper and butter. She’s 85 years old. I told her many times that it’s too salty… but at 85 years old, you won’t change her habits. But when I cook, I am careful with salt. (Patient 2, focus group #9)
	29	And some others say… well in this case, she [spouse] doesn’t want to have anything to do with it and he makes his own food and she makes her own food. (PR1MaC nurse, interview #4)

### Positive effects of the intervention

These perceived positive effects were grouped into 6 major themes: *awareness*, *improved knowledge*, *improved motivation and empowerment, adoption of healthy behaviours*, *improvement of health status* and *improvement of quality of life*. Among these themes, the first three are identified as intermediate effects, given that they contribute to the achievement of the three others, considered as final effects.

#### Awareness

The intervention contributed to an increased awareness among patients who had never been mobilized before in taking charge of their health (Table 2, quotes 1–5). Patients were aware of the importance of adopting and maintaining healthy lifestyle habits.

#### Improved motivation and empowerment

The program contributed to some patients becoming motivated and empowered to undertake a healthy shift in their lives and to change their lifestyle habits. For example, the program provided some patients with the motivation to stop smoking, and several patients to lose weight by adopting a better diet, to engage in physical activity and to resume monitoring their chronic diseases. In other cases, patients who had initiated changes in their lifestyle habits before the program acquired the motivation to maintain their positive health behaviours (Table 2, quotes 6, 7).

#### Improved knowledge

The program improved patient’s knowledge about the management of their chronic diseases. Many patients reported that they received advice and acquired tools to quit smoking, to adequately use their inhaler, to self-inject their insulin and take care of their diabetic foot, to eat better, to participate in physical activity and to manage their medication. The program also facilitated the acquisition of basic knowledge for patients never mobilized before. In other cases, the program allowed patients to deepen knowledge they had gained elsewhere (Table 2, quotes 8–10).

#### Adoption of healthy behaviour

Improved awareness, together with higher levels of knowledge, motivation and empowerment, had led patients to take charge of their health by making specific changes in their lifestyle habits.

Several participants stated that patients changed their dietary habits following the intervention. Patients reported that they had begun to read labels when shopping for groceries, to take more time to prepare meals, to eat well, to eat more fruits and vegetables, and to reduce portions. Also, they decreased their intake of certain foods and beverages (Table 2, quote 11). For some patients that had already changed their lifestyle, the program allowed them to do further changes. In terms of physical activity, participants noted that patients began exercising or increased their level of activity (Table 2, quotes 12, 13). A patient who had never before been mobilized to change his health behaviours also reported a positive experience (Table 2, quote 14).

#### Improvement in health status

Adopting healthy behaviours allowed patients to see concrete effects on their health. The majority of patients who had changed their health habits during the program reported positive results on their health. In this regard, several participants noted that patients were able to maintain their weight or, they managed to lose weight and reduce their waist circumference. Other participants stated that patients had better control of their blood glucose and blood pressure (hypertension), that they had improved their lipid profiles or their exercise capacity and that overall they had stabilized their chronic condition (Table 2, quote 15).

Finally, according to one patient who was previously unsuccessful with a diet, the program allowed her to lose so much weight that she thought she managed to avoid bariatric surgery (Table 2, quote 16). .

#### Improvement in quality of life

Participants reported positive impacts on patients’ quality of life. They noted that patients who had improved the quality of their sleep and experienced increased daily energy. Other participants stated that patients felt better about themselves; they had more self-confidence and self-esteem since they participated in the program and decided to take care of their health by adopting healthy behaviours. They added that patients felt much calmer, less stressed and anxious and that their level of guilt had decreased significantly (Table 2, quotes 17, 18).

#### Positive effects on family members

Some effects mentioned above were also observed among patients’ family members. In addition, the nature and extent of the intervention’s effect varied depending on the involvement or resistance to change among family members. In some families, the intervention had turned into a family issue involving all members in nutrition or physical activities. Parents or spouses made changes in health behaviours for children, wives or husbands. One Pr1MaC professional also observed contagious interest for change within certain families in terms of physical activity. In some instances, family members asked permission to come to the follow-up meeting for themselves (Table 2, quotes 19, 20).

### Negative effects of the intervention

Participants also reported some negative impacts. Perceived loss of the beneficial effects of the intervention was the negative effects most frequently mentioned. According to some participants, patients needed a longer follow-up period to maintain their motivation and knowledge acquisition (Table 2, quote 21). Patients and family members also abounded in the same sense, as seen in two excerpts from a patient focus group (Table 2, quotes 22, 23). Some patients also reported a significant weight gain. This led them to stop participating in the program. Similarly, one family member said that she was convinced that her partner had also stopped participating in the program due to a gain in weight (Table 2, quotes 24, 25).

Another patient also said that his weight gain at the end of the program was due to the fact that the program had ended before he had acquired enough knowledge on dietary behaviours (Table 2, quote 26). Finally, the absence of impacts can also be considered as a negative effect. According to some participants, the intervention did not have any effect: nothing happened or it didn’t produce any change in their life (Table 2, quote 27).

The effects of the intervention seemed limited in some patients due to the fact that the members of their family were opposed to their change (Table 2, quote 28). In some families, the patient was left alone with the changes to make (Table 2, quote 29).

## Discussion

Overall the program intervention did positively impact patients and their family members. The intervention increased awareness and knowledge, and improved motivation and empowerment. It facilitated the adoption of health behaviours, improvement of health status and improvement of quality of life. However, negative effects were also reported among patients, including a loss of the beneficial effects of the intervention. Past experiences of patients in relation to behaviour changes, and support or resistance of the family modulated the effects of the program.

This qualitative evaluation also provided insight that strengthens the quantitative results [4]. Both qualitative and quantitative studies reported increased *awareness* and *knowledge* among patients, *improvement in self-management*, and the *adoption of healthy behaviours*, including increased fruit and vegetable consumption and physical activity, as well as *health status* and *improvement of quality of life*. Similarities between qualitative effects and quantitative effects reinforce the results. Moreover, the qualitative results complement the richness of the quantitative results. They show in detail how these quantitative results are expressed by the patients and their family members in terms of life experience and perspective. The other positive effects identified in the qualitative evaluation (*improved motivation and empowerment*, and *improvement of health status*) were not measured in the quantitative analysis of program. Thus, they enrich the results of the research of program as a whole.

These findings are consistent with what was reported in the literature on patients with chronic conditions, suggesting that the integration and application of components of the Chronic Care Model in primary care organizations can improve patient outcomes, and should be supported [3, 11–13]. Our findings support the idea that CDPM programs involving interdisciplinary teams working with patients and their family members may represent an effective intervention strategy to respond to the complex needs of the patients seen in primary care. Our results are particularly interesting because they come from a program targeting several chronic conditions, unlike other programs also implemented in primary care, but single disease-oriented [14–23]. The qualitative evaluation indicates that the positive effects on patients were relatively different depending on their motivation when they entered the program [24]. All positive effects identified were observed in patients who had never before been mobilized to adopt healthy behaviours. Several positive effects were also observed among patients who had initiated changes in their health behaviours before the program. The program led them to reinforce their healthy habits or to adopt new ones. For patients in the maintenance phase, the effect of the program was an improvement in knowledge and motivation to persevere. The program helped patients who were not successful in the past with finding “motivation” and to regain control and adopt healthy behaviours.

Patients with multimorbidity represent a challenge for health care systems worldwide, given their complex needs. A majority of patients in this study had 3 or more chronic conditions. The findings both from the quantitative and qualitative evaluations clearly suggest that these patients may benefit from a CDPM program involving a team of professionals integrated in primary care at least in the short term. Maintenance of benefits on a long term basis still has to be demonstrated.

Our study has limitations. The limitations of the trial have already been published in the quantitative paper [4]. Self-reporting of outcomes is proned to desirability bias that may not be completely prevented but the use of several participants types (patients, family members and professionals). In addition, with one patient recruited out of four that were solicited, it is possible that patients perceiving positive results may have been more inclined to participate to the qualitative evaluation. Focus groups may be seen as a barrier for patients to express their feeling about the intervention. However, the experience of the interviewer was an asset to build trust and to encourage participation. The small number of patient family members who participated in the evaluation may also limit our comprehensive understanding of the effects of the program on family members. There is a need for further studies on family members’ involvement in CDPM programs.

Despite those limitations, this study adds to our understanding of the potential impact of CDPM programs integrated into primary care and not targeting one specific condition or risk factor. This approach is certainly in accordance with the philosophy of generalism that characterizes primary care. PR1MaC was a demonstration project that could inspire further development. Indeed, it provides attractive avenues that could improve the impact of integrated CDPM programs. This study where the intervention was carried out over a three-month period, supports the development of longer interventions. Although it is not clear how long such intervention should be, it remains that patients need time to make significant changes particularly relating to health behaviour. Further studies should also address long-term sustainability of benefits to the patients.

## Conclusion

These qualitative findings complement the results of the PR1MaC trial and identified positive effects beyond those reported in the quantitative study. Integrating chronic disease prevention and management programs in primary care focusing on several chronic conditions is feasible and associated with positive outcomes for the patients and to some extent to their family members. Maintaining the outcomes over time is a challenge that has yet to be addressed.

## Additional file

Additional file 1: Characteristics of the interventions This additional file describes details of the interventions. (DOCX 17 kb)

## Abbreviations

CDPM: Chronic disease prevention and management
COREQ: Consolidated Criteria for Reporting Qualitative Research
SD: Standard deviation
