# Supplementary material for: Integration of chronic disease prevention and management services into primary care (PR1MaC): findings from an embedded qualitative study
Source: BMC Fam Pract. 2019 Jan 9;20:7. doi: 10.1186/s12875-018-0898-z (PMC6325817; doi:10.1186/s12875-018-0898-z)
Supplement: Supplementary file 1 — Characteristics of the interventions This additional file describes details of the interventions. (DOCX 17 kb) [file 12875_2018_898_MOESM1_ESM.docx]

**Additional file 1** Characteristics of the interventions

| **CDPM training** | Theoretical training of the interdisciplinary team on:   - Motivational interviewing - Function of the respiratory system - Function of the cardiovascular system - Diabetes - Risk factors - Existing CDPM services   Practical training:  Three-week mentoring in specialized CDPM services facilities. |
| --- | --- |
| **Preliminary clinical evaluation of the patients** | The clinical evaluation of patients included:   - Anthropometric characteristics - Medical history - Medication - Functions (respiratory, cardiovascular, endocrine, gastro-intestinal) - Lifestyle habits and risk factors - Patient’s concerns and objectives - Previous interventions (nutrition, physical activity, respiratory, smoking cessation), - Recent changes (weight, alcohol consumption) |
| **Disciplines involved in the intervention** | The individualized intervention based on a referral from a family physician or nurse, were provided by professionals among the following disciplines:   - Nursing - Physical activity therapy - Nutrition - Respiratory therapy - Smoking cessation therapy |
| **Intervention components** | The individualized interventions consisted of several components among the following:   - Self-management support - Education on diseases (diabetes, COPD, asthma, cardiovascular, etc) - Education on risk factors (pre-diabetes, high blood pressure, dyslipidemias, obesity, physical inactivity, smoking) - Counseling on medication - Motivational interviewing - Education about nutrition - Education about physical activity - Counseling on smoking cessation |
| **Tools and support material** | Each intervention was supported by print and other material to ensure that patient engagement was maintained even between the visits. These include documents among the following:   - Chronic disease management - Asthma, COPD - Diabetes - Cardiovascular - Metabolic syndrome - Hypo/hypertension - Tools for smoking cessation - Stress management - Blood pressure monitoring journal - Personal objectives journal - Physical activity journal |
| **Communication & coordination** | The CDPM professionals were integrated into the primary care practices which enhanced communication with primary care physicians, nurses and staff. A clinical coordinator ensured optimal communication and transition of care between the project team, the primary care professionals and specialized services. Special attention was given to the distinction of tasks fulfilled by CDPM professionals and tasks fulfilled by primary care nurses. |
| **Integration** | Prior to the implementation of interventions, a pre-implementation evaluation was conducted to identify the needs for CDPM services and the contextual factors of the participating PC clinics in the follow-up of CD patients. The pre-implementation evaluation of the project promoted the sharing of a common positive vision of an intervention that focuses on prevention, earlier support for patients in the course of their disease, interprofessional collaboration, services integration, motivational interviewing and self-management support. |
